# Opto‐Thermal‐Tension Mediated Precision Large‐Scale Particle Manipulation and Flexible Patterning

**DOI:** 10.1002/advs.202405211

**Published:** 2024-07-25

**Authors:** Ziyi He, Jianyun Xiong, Yang Shi, Guoshuai Zhu, Xing Li, Ting Pan, Baojun Li, Hongbao Xin

**Affiliations:** ^1^ Guangdong Provincial Key Laboratory of Nanophotonic Manipulation Institute of Nanophotonics Jinan University Guangzhou 511443 China

**Keywords:** optical manipulation, opto‐thermal‐tension effect, particle patterning

## Abstract

Large‐scale particle manipulation with single‐particle precision and further flexible patterning into functional structures is of huge potentials in many fields including bio‐optoelectronic sensing, colloidal lithography, and wearable devices. However, it is very challenging for the precision manipulation and flexible patterning of particles on complicated curved and functional substrates. In this work, opto‐thermal‐tension (OTT) mediated precision large‐scale particle manipulation and flexible patterning based on soap film are reported. Flexible manipulation and subsequent patterning of particles with single‐particle resolution is realized by optothermal regulated surface tension on soap films. Reconfigurable patterning of particle structures with different shapes as well as large‐scale ordered structures (up to 2000 particles) with particle sizes spanning two orders of magnitude (0.5–20 µm) is realized using this OTT mediation method. Importantly, due to the high flexibility of soap films, the patterned large‐scale particle structures can be non‐destructively transferred to curved and rough substrates, including rough iron pipe surface, leaf and skin surface. This OTT mediated method provides a new method for precision large‐scale particle manipulation and flexible patterning with high versatility on complicated functional substrates, with great potentials for optoelectronic and biophotonic sensing and wearable device design on different curved and rough functional substrates.

## Graphical abstract

An opto‐thermal‐tension (OTT) mediated manipulation method is reported for precise large‐scale particle manipulation and flexible patterning on soap film. Reconfigurable patterning of particle structures with different shapes as well as large‐scale ordered structures is realized. Due to the high flexibility of soap films, the patterned large‐scale particle structures can be non‐destructively transferred to curved and rough substrates.

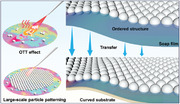

## 1 Introduction

Large‐scale manipulation and patterning of particles into functional ordered structures has attracted great attention in various fields due to their diversity of optoelectronic properties.^[^ ^1^, ^2^ ^]^ Such particle patterns have been widely used in different areas such as cargo transport,^[^ ^3^ ^]^ conductive films,^[^ ^4^ ^]^ electrochemical sensing,^[^ ^5^, ^6^ ^]^ biophotonic sensing,^[^ ^7^ ^]^ and wearable devices.^[^ ^8^, ^9^ ^]^ Due to the wide range applications of ordered particle structures, a variety of feasible assembly and patterning strategies have been explored, including spin coating,^[^ ^10^, ^11^, ^12^ ^]^ dip coating,^[^ ^13^, ^14^, ^15^ ^]^ drop coating,^[^ ^16^, ^17^ ^]^ and electrophoretic deposition.^[^ ^18^, ^19^, ^20^ ^]^ However, the above methods are limited for particle assembly on smooth and planar solid substrates.^[^ ^21^, ^22^ ^]^ For the assembly of particle structures on rough and curved substrates, researchers explored the self‐assembly method via air/liquid interface mediation and subsequently transferred the assembled particle structures to a curved and rough substrate.^[^ ^23^, ^24^, ^25^, ^26^, ^27^ ^]^ Although the above methods can realize the assembly and patterning of large‐scale particle structures, the patterning resolution is limited, and they are unable to precisely manipulate single or small clusters of particles. Therefore, there will inevitably exist defects in the large‐scale ordered particle structures obtained by these methods. In addition, the assembly of particle structures with specific patterns and shapes necessitates the assistance of templates, which limits the further applications of specific patterns on complicated functional substrates.

On the other hand, optical tweezers‐based optical manipulation technique has been widely used for particle manipulation.^[^ ^28^ ^]^ Optical tweezers are originated from the use of focused laser beams to precisely manipulate particles. This method utilizes the momentum interaction between the focused laser beam and target particles to achieve the precise control of a single particle. Different particles, from dielectric particles to biological cells, can be precisely manipulated using optical tweezers.^[^ ^29^ ^]^ The trapped particles can further be pattered into designated structures via either precisely manipulating the single laser focus or designing trapping platforms with multiple trapping points such as structured light beams,^[^ ^30^ ^]^ holographic optical tweezers,^[^ ^31^ ^]^ plasmonic tweezers,^[^ ^32^ ^]^ and photonic crystal tweezers.^[^ ^33^ ^]^ However, due to limit of manipulation throughput and flexibility, it is still a great challenge for the precision large‐scale manipulation and patterning using conventional optical tweezers‐based manipulation techniques. In addition to optical tweezers, other tweezers‐based manipulation methods have also been used for particle patterning, for example, optoelectronic tweezers,^[^ ^34^ ^]^ and acoustic tweezers.^[^ ^35^ ^]^ These tweezer forms have improved manipulation throughput. However, the patterning flexibility is still limited.

**Figure 1:**
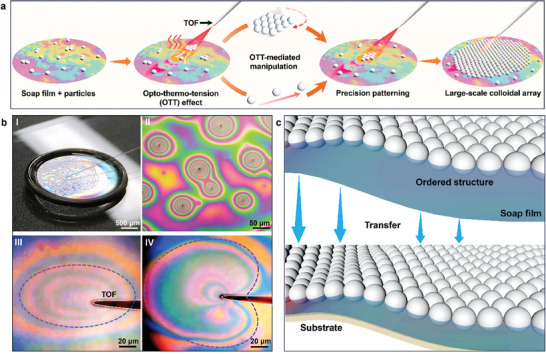
Overview of OTT‐mediated large‐scale particle manipulation and flexible patterning on soap film. a) Schematic diagram of the process of OTT‐mediated particle manipulation and patterning on soap film. b) Experimental images of soap films for OTT‐mediated particle manipulation and patterning. I) Photograph showing the experimental setup with soap film with 3‐µm PS particle dispersed, the soap film was supported by a rubber ring and with a TOF placed above. II) Microscopic images showing interference rings surrounding single particles or clusters on the soap film. III, IV) Microscopic images showing the change of interference ring by controlling the movement of the TOF tip due to optothermal effect. c) Schematic illustration showing transfer of assembled large‐scale ordered structures from soap film onto rough and curved substrate.

In this work, we report opto‐thermal‐tension (OTT) mediated particle manipulation and flexible patterning on soap film that can be transferred to rough and curved substrates. Such OTT method enables the precise manipulation and patterning of single particle or small particle clusters, and finally large‐scale particle arrays on a flexible soap film as the assembly substrate (**Figure** **1**a). We realized the patterning of particle structures with specific shapes on the flexible soap film via the high‐precision control of optothermal effect‐mediated surface tension via light irradiation using a tapered optical fiber probe (TOF). By OTT‐mediated assembly of the patterned small particle modules, larger‐scale particle crystals with particle size spanning two orders of magnitude (from 500 to 20 µm) were assembled on soap film. Finally, the large‐scale particle crystals on the soap film were non‐destructively transferred to rough and curved substrates such as leaf, skin and iron pipe. This OTT‐mediated particle manipulation and flexible patterning on soap film provides a new method for the flexible patterning of large‐scale particle structures with high precision, providing the possibility to expand the application range of large‐scale particle structures on rough and curved functional substrates, such as optoelectronic sensing, biophotonic sensing, as well as design of wearable devices.

## 2 Results

### 2.1 OTT Mediated Large‐Scale Particle Manipulation and Flexible Patterning

To realize the large‐scale particle manipulation and flexible patterning with high precision and controllability, soap films were used as the assembly substrate because of the high flexibility and strong plasticity. Figure 1a schematically shows the process of the OTT‐mediated particle manipulation and flexible patterning on soap films via precision particle manipulation regulated by the optothermal effect‐induced surface tension.^[^ ^36^, ^37^, ^38^ ^]^ In the experiments, a soap film (thickness: 100–200 nm) with microparticle dispersed was obtained from a particle‐soapy solution and was supported by a rubber ring (Figure 1b‐I, see Experimental Section for the details of preparation). With microparticles dispersed on the soap film, the distance between the double layers of the soap film was changed. Due to the equal‐thickness interference, interference rings with rainbow colors appeared around the single particle or particle cluster on the soap film, as shown in Figure 1b‐II. A TOF was placed above the surface of a soap film with a distance of about 20 µm (Figure 1b‐I, Figures S1 and S2, Supporting Information, details see the Experimental Section). With a laser beam at a wavelength of 808 nm (optical power: 20–60 mW) launched into the TOF, due to the light absorption‐induced optothermal effect of the soap film, the surface tension of the soap film was reduced at the light irradiation position, resulting in the localized OTT effect. The thickness of the soap film around the irradiation position will shrink due to the optothermal effect, and thus, change of the interference ring can be observed as shown in Figure 1b‐III,b‐IV (see Videos S1 and S2, Supporting Information). The gradient of surface tension can result in the motion of the microparticles and particle clusters on the soap film. This motion can be precisely regulated via the OTT effect by adjusting the distance between the TOF tip and the soap film surface and the angle at which laser irradiates the soap film surface, as shown in Figure S3 (Supporting Information). Using the OTT effect, the movement of individual particles as well as the rotation of particle clusters on the soap film can be precisely manipulated by moving the TOF, and large‐scale particle array can then be patterned by the assembly of individual particles or particle clusters. Eventually, large‐scale ordered structures with designated patterns can be formed on soap film with single particle precision. Importantly, due to the high flexibility of the soap film, the formed ordered structures can be transferred from the soap film to target rough and curved substrate, as shown in Figure 1c (details see Experimental Section).

### 2.2 OTT‐Mediated Precision Particle Manipulation and Patterning

**Figure 2:**
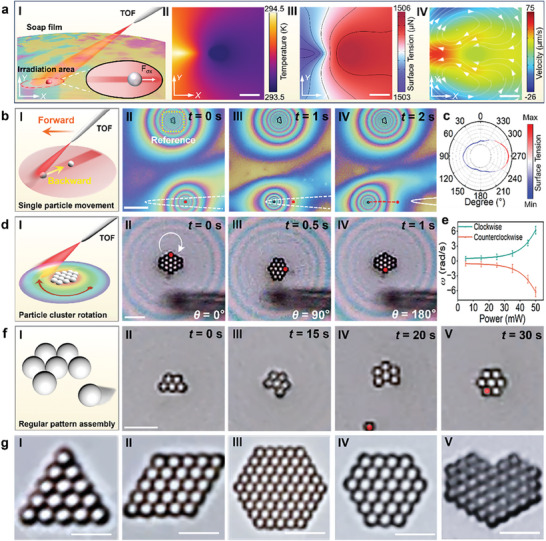
OTT‐mediated particle manipulation and patterning. a) Simulation of OTT effect on soap film. I) Schematic illustration showing laser irradiation from a TOF for particle manipulation on soap film. Inset shows the moving direction of a single particle along surface tension gradient in the irradiation area. Simulated results of II) temperature, III) surface tension, and IV) flow field on soap film with light irradiation. b) Single particle manipulation by surface tension gradient. I) Schematic showing the manipulation of a single particle backward with TOF moving forward. II–IV) Microscopic images showing single particle manipulation by the laser beam from the TOF. The yellow curve indicates a particle cluster which is in a stationary state as a reference. The red dot represents the initial position of the particle, the white dashed and solid curves represent the original and moving position of the TOF, respectively, and the red dotted line represents the trajectory of the particle during manipulation. c) Simulated surface tension around the particle. d) Rotation of particle cluster. I) Schematic showing the rotation of particle cluster. II–IV) Microscopic images showing the clockwise rotation of particle cluster. The red dots indicate a reference particle during rotation. e) Rotation speed as a function of laser power. f) OTT‐mediated assembly of a hexagonal pattern. I) Schematic of the assembly. II–IV) Microscopic images showing the assembly process. g) Assembled particle patterns with shapes of I) regular triangle, II) rhombus, III) regular hexagon, IV) diamond, and V) heart shape. Scale bars: 10 µm.

To show the mechanism and performance of OTT‐mediated particle manipulation and patterning, numerical simulation was first carried out (details see Supporting Information). As shown in **Figure** **2**a‐I, due to the inclined angle (15°) of the incident laser beam, the irradiation area on the soap film was an ellipse. The motion of particle in the irradiation area is affected by surface tension in all directions. As shown in the inset of Figure 2a‐I, the surface tension on both sides along the long axis of the irradiation ellipse is symmetric, and thus the surface tension is counterbalanced on the particle. Therefore, the effective surface tension that can affect the particle is along the long axis of the irradiation ellipse. As shown in Figure 2a‐II, due to light absorption of the soap film (10^3^ cm^−1^ at 808‐nm wavelength laser beam),^[^ ^39^ ^]^ localized temperature change can be induced at the light irradiation region with an optical power of 20 mW irradiation (size of laser spot: an ellipse with a major axis length of 60 µm and a minor axis length of 20 µm). The localized temperature increase can increase the thermal motion of the molecules in the soap film. Compared with the lower temperature locations, the distance between molecules in soap film is then increased, so the mutual attraction between molecules is decreased, resulting in the decrease of surface tension.^[^ ^40^, ^41^ ^]^ The surface tension coefficient is estimated to be 24 mN m^−1^, the surface tension change rate is 0.036 mN m^−1^ K^−1^,^[^ ^42^ ^]^ and the viscosity coefficient is 2.2 × 10^5^ Pa s. Figure 2a‐III shows the simulated localized surface tension. The simulated surface tension is symmetric along the *y*‐axis direction (see Figure S4a, Supporting Information), for particle located along the *x*‐axis (*y* = 0), the resultant surface tension in the *y* direction is 0. While along the *x*‐axis direction, there is temperature gradient, and therefore, there also exists surface tension gradient exerted on particle along the *x*‐axis direction (*y* = 0) (see Figure S4b, Supporting Information). As a result, the particle can be transferred along the direction of resultant force in the *x*‐axis. Therefore, the particle can be controlled along the *x*‐axis direction by moving the TOF. In addition, for particles that are not on the symmetry axis of the light irradiation area, the resultant force on the *y*‐axis is not 0, so the particle will be pulled toward the location where the surface tension is high. Through the simulation in Figure 2a‐IV, due to the surface tension induced by optothermal effect, a symmetrical flow field will appear on both sides of the symmetrical axis of the irradiation area, also known as the Marangoni flow field,^[^ ^43^, ^44^ ^]^ which can be used for the controllable rotation of particles. Combing the particle manipulation along the symmetrical axis and rotation on both sides, particles can be precisely manipulated by the OTT effect, and further for flexible patterning on soap film.

Figure 2b shows the precision manipulation of a single particle. By moving the TOF above a target particle forward, the particle will be manipulated backward (Figure 2b‐I). Figure 2b‐II,b‐IV shows the manipulation process of a single particle (polystyrene (PS) particle, diameter: 3 µm). The TOF with 808 nm laser launched (20 mW) was controlled to move above a particle on the soap film from right to left along the *x*‐axis direction (see Video S3, Supporting Information). During the TOF moving, the optothermal effect at the directly irradiated position resulted in the temperature on the left side of the particle higher than that on the right side. The surface tension on the left side of the particle is smaller than that on the right side, as shown in Figure 2c and Figure S5 (Supporting Information). Thus, the particle was moved rightward along the *x*‐axis with a distance of 25 µm (*t* = 2 s) under the pulling of the surface tension gradient. When the laser was turned off, the temperature gradient on the soap film quickly decreased, and the temperature gradient‐induced surface tension gradient gradually decreased to 0, eventually causing the particles to stop moving. During the manipulation, the displacement precision is measured to be about 0.45 µm for a 2‐µm PS particle (details see in Figure S6, Supporting Information). This method can also be used for the manipulation of individual particles in a multi‐particle environment, where the particles are in a discrete state. To realize this, the particles must be kept at a sufficient distance between each other to avoid aggregation caused by tension attraction. This effective distance range is about 10–15 µm and can be controlled by adjusting the laser power as well as the distance between the TOF and the soap film. With the laser power smaller than 5 mW, and the height is 10 µm above the soap film, the effective manipulation range can be reduced to about an ellipse with the length of the long axis of about 10 µm. With this manipulation range, individual particles in a multi‐particle environment can then be precisely manipulated. As shown in Figure S7 (Supporting Information), by using this method, we can manipulate the individual particles to form a discrete triangle, hollow parallelogram, and solid parallelogram. However, when multiple particles are already formed into stable close‐packed dense structures, individual particles within the multi‐particle structures cannot be manipulated. Instead, we can manipulate the multi‐particles dense structures/clusters. Figure 2d‐I schematically shows the precision rotation of a particle cluster. During the rotation process, the TOF tip was kept still above the interference ring around the particle cluster. The particle cluster was controlled to rotate by OTT‐induced Marangoni flow field (shown in Figure 2a‐IV and Video S4, Supporting Information). As an example, Figure 2d‐II,d‐IV shows the clockwise rotation of a hexagon particle cluster (composed of 14 PS particles, diameter: 3 µm). The fiber tip was placed with a distance of 10 µm to the particle cluster in the *y*‐direction. The average rotation speed was 1 rad s^−1^ at the optical power of 45 mW. The rotation direction can be controlled simply by placing the TOF at the opposite side of the cluster. As shown in Figure 2e, as the laser power was increased, the rotation speed of the particle cluster was increased exponentially.

Combing the precision migration and rotation of single particle as well as particle cluster by OTT effect, assembly of particles with designated patterns on soap film can then be realized. As an example, Figure 2f shows the patterning of a regular hexagon with 7 PS particles (diameter: 3 µm) by cooperatively manipulating single particles or rotating assembled particle cluster. Similarly, particles with other different patterns can be assembled by manipulating either a single particle or sub‐clusters. Figures S8 and S9 (Supporting Information) show the detailed process for the assembly of different particle patterns. Figure 2g shows some of the assembled particle patterns, such as regular triangle, rhombus, regular hexagons, and creative patterns like diamond shape and heart shape. In addition to the close‐packed structures, non‐close‐packed structures can also be patterned on soap film (Figure S10, Supporting Information). It should be noted that although these non‐close‐packed structures can be formed, these structures are less stable than the close‐packed structures on soap film. The reason is that surface tension on soap film can result in the contraction on patterned particles, and these non‐close‐packed structures on the soap film will gradually be squeezed into the close‐packed dense structures. Further experimental results show that our method can also be used for the hybrid patterning of particles with different compositions. As some examples, Figure S11a (Supporting Information) shows the hybrid patterning using two different sized spherical PS particles with diameters of 3 and 2 µm on soap film, while Figure S11b (Supporting Information) shows the hybrid patterning using three different sized PS particles with diameters of 3, 2, and 1 µm. During the hybrid patterning of different sized particles, the largest 3 µm particles are patterned in the center, and smaller ones are packed around the central larger particles. In addition to the spherical microparticles, our method can also be used for the patterning of other shaped particles, including biological particles. For example, Figure S11c‐I,c‐II shows the patterning of square‐shaped silica particles, while Figure S11c‐III,c‐IV shows the patterning of spindle‐shaped *Phaeodactylum tricornutum* Bohlin, and Figure S11c‐V shows the patterning of rod‐shaped *E. coli* bacteria.

### 2.3 Large‐Scale Patterning and Deformation of Particle Structures on Soap Films

**Figure 3:**
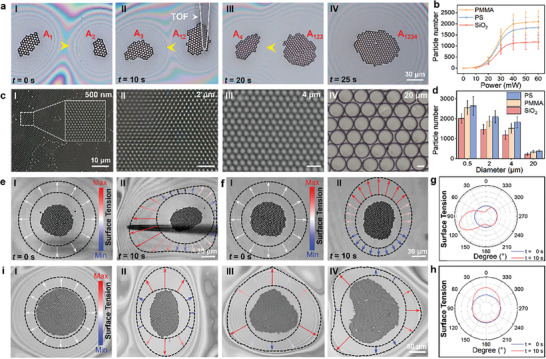
Large‐scale patterning and deformation of ordered particle structures on soap films. a) Microscopic images showing assembly of large‐scale particle structures by manipulating small modules, yellow arrows indicate the moving direction of the central module. b) Particle number in the final stable particle structures as a function of laser power. c) Large‐scale particle structures assembled with PS particles of I) 500 nm, II) 2 µm, III) 4 µm, and IV) 20 µm. d) The number of particles in the final assembled stable particle structures as a function of particle size. Deformation of circular particle patterns into ellipse by stretching along e) *x*‐axis and f) *y*‐axis direction. The black curved dotted lines represent the outer and inner shape of the interference ring, the colored arrows represent the surface tension around the particle pattern. The surface tension diagram around pattern before and after OTT mediation in the g) *x* and h) *y* direction. i) Examples of formed particle pattern with different shapes of I) circular, II) elliptical, III) triangular IV) heart shaped. The black curved dotted line indicates the shape of the inner interference ring and the outer interference ring. The colored arrows indicate the surface tension around the pattern.

After the high‐precision manipulation and regular patterning of particles on soap film through OTT effect, large‐scale particle structures and crystals can be further formed by assembly of previous assembled particle modules, similar to the game of Tetris. As an example, **Figure** **3**a shows the assembly of a large particle module with 245 PS particles (diameter: 3 µm, optical power: 30 mW). For large‐scale particle assemble, a central particle module was first designated (indicated as A_1_ with particle number of 45 in Figure 3a‐I), and then small particle modules near the central module were controlled to move toward the central module by the OTT effect, similar to the manipulation of a single particle or small particle cluster. Module A_2_ was then moved toward A_1_ and combined into a new module A_12_ (particle number: 85, Figure 3a‐II) under the attraction of intermolecular force. Similarly, particle modules A_123_ and A_1234_ with respective particle number of 163 and 245 were assembled, as respectively shown in Figure 3a‐III,a‐IV. It should be noted that after small particle modules near the central module were assembled and merged with the central module, the new central module composed of hundreds of particles should be moved to find new particle modules which were far away from the central module. Because of the increasing size of the central module, a larger surface tension force is necessary to move the center module. The laser power was then increased to increase the surface tension to move the large particle module for large‐scale particle structure assembly. As shown in Figure 3b, as the laser power increases, the final assembled particle number is gradually increased. However, due to the limited bearing capacity of the soap film, the particle number in the final assembled large‐scale particle structures and crystals that can be maintain stable (with a maintaining time longer than 5 min) is limited. As shown in Figure 3b, the final particle number is about 2100, 1830, and 1180 for Polymethyl Methacrylate (PMMA), PS, and Silicon dioxide (SiO_2_) (diameter: 3 µm). The difference in particle number for particles with different materials resulted from the different density. Importantly, the large‐scale assembly of ordered crystals on soap film is capable for particles with sizes spanning two orders of magnitude (0.5–20 µm). Figure 3c shows some examples of the assembled crystals based on PS particles with size of 500 nm, 2, 4, and 20 µm. During the assembly, the generated defects can also be repaired using our method. Due to the fluidity of the soap film, by adjust the position of the TOF, we can control the localized movement of the colloidal particles within the assembled particle structures by the OTT effect for particle reordering. Under the squeezing effect of the surface tension of the soap film, neighboring particles can move toward the central gap, thereby filling the defect in the colloidal particle structures. As some examples, Figure S12 (Supporting Information) shows the repairing of defects within the patterned particle structures. Both a single point defect (vacancy) and multiple defects can be repaired. Figure 3d shows the final particle number in the formed stable crystals with different particles on the soap film. Among them, the number of particles in the array composed of 500 nm, 2 µm, and 4 µm PS particles reached 2000. For the assembly of 20 µm particle arrays, the number was only about three hundred due to the limitation of the bearing capacity of the soap film.

After large‐scale particle crystal assembly, the deformation capability of the particle structure is also very important for further functionalization. As the size of the structure is gradually increased to more than 400, we can clearly observe that the shape of the interference ring surrounding the assembled particle structure is changed with the shape of the structure profile, indicating the change of soap film thickness. As mentioned in Figure 1b‐III,b‐IV, the soap film thickness can be changed, and the interference rings on the soap film can then be deformed into other shapes under the regulation of optothermal effect. This change can further result in the deformation of assembled structures due to the OTT effect. Therefore, we can regulate the deformation of the assembled structures using our OTT method. As some examples, Figure 3e shows the stretching of a formed particle structure (composed of PS particles, diameter: 3 µm) from a circular shape to an ellipse along the *x*‐axis direction. The fiber tip was controlled to move along the *x*‐axis and to squeeze the interference ring on the left side of the structure. The original circular interference ring was then gradually changed to an ellipse shape. The uneven spacing of interference ring indicates that the thickness of the soap film around the particle structure became uneven, therefore, the surface tension gradient was not equal, as shown in Figure 3g. Unequal surface tension in different directions can stretch the particle array in the direction with high surface tension, thereby achieving the controlled deformation of the large‐scale particle structure. At *t* = 10 s, the original circular structure was deformed into an ellipse shape with the long axis along the *x* direction. Similarly, as shown in Figure 3f, by controlling the fiber tip to move along the *y*‐axis and to squeeze the interference ring in the *y*‐axis direction, circular shaped structure was deformed into an ellipse along the *y*‐axis direction under the stretching of surface tension gradient force along the *y* direction (Figure 3h). By using OTT effect to regulate the surface tension at different directions around the formed structure, as shown in Figure S13 (Supporting Information), particle structures with different shapes can be regulated. As some examples, Figure 3i shows the formed structures with different shapes of circular, elliptical, triangular, and heart‐shaped pattern.

### 2.4 Transfer of Ordered Particle Crystals onto Curved and Rough Substrates

**Figure 4:**
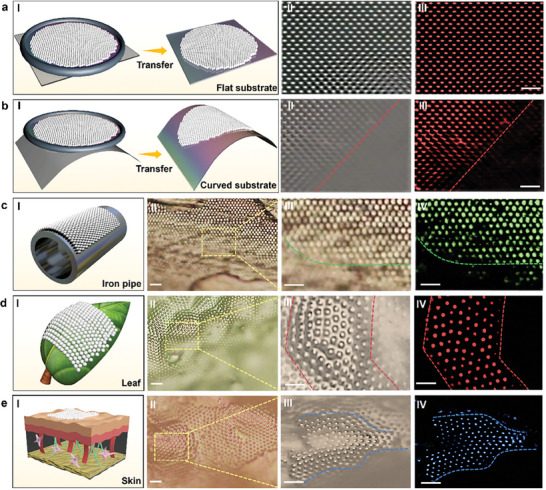
Transfer of particle crystals from soap films onto different substrates. a) Flat substrate. I) Schematic showing the transfer of large‐scale crystal onto flat PDMS substrate. II) Bright field microscopic image and III) fluorescence image showing the large‐scale crystal on PDMS after transfer. b) Curved substrate. I) Schematic showing transfer of large‐scale crystal onto curved PDMS substrate. II) Bright field microscopic image and III) fluorescence image showing the large‐scale crystal on curved PDMS substrate. Transfer of large‐scale crystal onto different rough and curved substrates of c) iron pipe surface, d) leaf, and e) human skin. I) Schematic diagram of the transfer. II) Bright field microscopic image showing the transferred crystal the substrates. Enlarge view of the crystal under III) bright field microscopic image and IV) fluorescence image. Scale bars: 10 µm.

Flexible patterning of ordered particle structures on curved and rough functional substrates is very important for further use in different applications, such as optoelectronic sensing, biosensing, and wearable devices. Due to the high stability, reconfigurability and flexibility of the formed ordered particle structures on soap film, we can transfer the assembled particle structures and crystals from the soap film to different substrates, including curved and rough ones. Because of the high flexibility of soap films, the soap film can be easily adhered to different substrates by viscosity of the soap film, and the formed structures and crystals can then be transferred onto the substrate without damage along with the soap film (details of the transfer see in the Experimental Section). **Figure** **4**a shows that a large‐scale ordered particle crystal (fluorescent PS particles, diameter: 3 µm, particle number about 380) was transferred onto a flat Polydimethylsiloxane (PDMS) substrate. From both the bright field and fluorescent microscopic images, we can see that the particle crystal was maintained stable after the transfer. In addition to the transfer onto flat substrate, as shown in Figure 4b, the formed crystal can also be transferred to the curved PDMS substrate using the same method. The transferred particle structure was still maintained an orderly and compact arrangement on the curved substrate.

In addition to the changeable PDMS substrate, for many other sensing applications, however, the substrate is often in a rough situation with many sensors and detection components on the surface. Assembly of ordered particle structures on such substrates is often very challenging. Importantly, the formed particle crystals on soap film can be easily transferred onto different complicated rough and curved substrates, which can be further used as a coating layer. Figure 4c shows the transfer of particle crystal to a rough and sharply curved iron pipe surface. After the transfer, the integrity of the crystal was still highly maintained. In addition, such transfer method can further be used for different biologicals substrates. For example, Figure 4d,e shows the transfer of particle crystal from soap film to the surface of a leaf and skin, respectively. From both the bright field and fluorescent microscopic images, it can be seen that the particle crystal can be easily transferred and perfectly attached to the highly undulating surface of leaf and skin. The transfer of particle structures onto different substrates, particular the sharply curved iron pipe surface and skin, provides many possibilities of ordered particle structures on different substrates for further applications, such as optoelectronic sensing, biophotonic sensing, as well as wearable devices. In particular, since a spherical particle can serve as a microlens to generate photonic nanojet for effective light focusing. After transferring onto different substrates, the large‐scale particle patterns can then serve as microlens array. This microlens array can generate large‐scale photonic nanojet array for effective light focusing, as show in Figure S14 (Supporting Information). This feature suggests that the patterned structures can serve as photonic platforms with different biophotonic applications based on the focused light beam and enhanced optical signals, for example, for bio‐molecular sensing and detection, as well as large‐scale bio‐particle trapping and manipulation.

## 3 Discussion

In summary, we report an OTT‐mediated method for precision large‐scale particle manipulation and flexible patterning on soap film. This particle manipulation and patterning method utilizes the optothermal effect‐induced surface tension gradient change on highly flexible soap film, which can accurately control the motion and rotation of single particle as well as particle clusters. Particles can be precisely assembled into different designated patterns with different shapes using this OTT method. Large‐scale ordered particle structures and crystals with colloidal particle number up to 2000 can be easily formed with high deformation and reconfiguration capability, with the sizes of particles that can be assembled spanning two orders of magnitude (0.5–20 µm). Importantly, the formed large‐scale particle structures on the soap film can be easily and non‐destructively transferred to different curved and rough substrates, such as PDMS and iron pipes, as well as soft biological substrates, such as leaf and skin.

Many different methods have been used for particle manipulation, assembly and patterning, for example, thermophoresis, where particles can move along a temperature gradient field,^[^ ^45^ ^]^ and electrophoresis, particles can be precisely manipulated in electric fields.^[^ ^46^ ^]^ Both thermophoresis and electrophoresis have been widely used for particle trapping, manipulation, assembly and patterning. However, for thermophoresis, the ordered large‐scale assembly and patterning with high throughput is still challenging; while for electrophoresis, the ordered patterning generally necessitates predesigned substrates with ordered electrode arrays. Compared with particle manipulation using thermophoresis or electrophoresis, our OTT mediated manipulation on a soap film exhibits higher flexibility for both single particle manipulation and large‐scale particle patterning. In addition, different on‐surface optothermal manipulation tools have served as excellent techniques for the precision manipulation and patterning of particles on surface. For example, Li et al. demonstrated an optothermal‐gated photon nudging (OPN) technique for the precision manipulation of nanoparticles on solid substrates.^[^ ^47^, ^48^ ^]^ In the OPN technique, a thin surfactant layer is introduced between particles and the solid substrate and served as optothermal gate to modify particle‐substrate interfacial interactions. Strong light‐response particles can then be precisely manipulated by optical scattering force after the surrounding surfactant being heated and phase transited via the localized optical heating of the particle. Such OPN technique enables the precision manipulation of particles with different sizes and materials, and the particles can be easily patterned into different functional structures on solid surface. Compared with the OPN technique, our OTT mediated manipulation method is based on a soap film and has no restrictions on the photothermal effect of the manipulation particles. Instead, in our OTT method, the photothermal effect resulted from the light absorption of soap film, and the particle is then manipulated and patterned by the surface tension on the soap film around the particle. Our OTT technique enables the precision large‐scale particle patterning. The flexibility of the soap film also enables the patterned particle structures to be transferred onto both flat and complex curved substrates, expanding the potential applications of the patterned large‐scale particle structures. This OTT‐mediated large‐scale particle manipulation method provides a new way for particle manipulation, and the flexible patterning and assembly particle structures on soap film provides a new platform for flexible ordered particle structure assembly. The non‐destructive transfer of ordered particle structures onto different substrates, particularly the curve and biological ones, provide new possibilities for further optoelectronic and biomedical applications, such as optoelectronic and biophotonic sensing, as well as wearable device design.

## 4 Experimental Section

### Preparation of Soap Film

Soap solution was first prepared using deionized water and glycerin in a ratio of 4:1. Sodium lauryl sulfate, dodecyl dimethyl amine oxide, alkylamines, and water‐soluble lanolin were then added with a final concentration of 11.9, 44.88, 48.24, and 5.3 mm, respectively. The mixed solution was stirred for 30 s at 100 rpm and extracted to a syringe to prepare for the next step of operation. A filter membrane with a mesh size of 500 nm (purchased from Fumei Technology, Xiamen, China) was mounted on the syringe. The syringe was pushed slowly to allow the mixed solution to drop out of the filter membrane. The purpose was to fully filter the bubbles generated during the process of shaking and mixing, as well as the impurities in the mixed solution. 10 µL of 3‐µm PS solution (purchased from Wuhan Huake Microtechnology Co., Ltd, Shanghai, China) with a concentration of 0.1 mg mL^−1^ was added to 2 mL of soap solution and mixed thoroughly, and suspension with 3‐µm PS particles was then obtained. A rubber ring with diameter of 2.5 µm (purchased from Haohuan Sealing Products Co., Ltd, Guangdong, China) was placed in the suspension, and the suspension was added continuously until the rubber ring was completely submerged. After immersing for 10 s, the rubber ring was slowly pulled out of the suspension, and a stable soap film was obtained in the central part of the rubber ring.

### Preparation of Tapered Optical Fiber

Tapered optical fiber (TOF) was made by a flame‐heating commercial single‐mode optical fiber (connector type: FC/PC, core diameter: 9 µm, cladding diameter: 125 µm). To protect the thin, fragile optical fiber while controlling the optical fiber using the optical fiber manipulator, the optical fiber was wrapped in iron pipe (inner diameter: ≈1 mm, thickness: ≈0.2 mm, length: ≈150 mm) before the fiber was tapered. Before the optical fiber was tapered, the protective outer jacket layer, coating and cladding layer of the optical fiber were stripped off by fiber stripper in sequence, leaving the fiber core with ≈120 mm long and 125 µm in diameter exposed. Then, two tweezers were used to fix the exposed optical fiber on both sides, and the middle part of the fixed optical fiber was placed in the outer flame of the alcohol lamp for heating. After heating for about 30–60 s, the temperature of the heating area with a length of 2–3 µm reached the melting point of the fiber. At this time, the optical fiber was stretched to both sides at a speed of about 0.3 mm s^−1^ until the length of the tapered area in the middle reached about 3 cm. Then a faster speed of 2 mm s^−1^ was applied, causing the fiber to break and leave the flame quickly. When the fiber in the molten state was broken, a tapered end with a parabolic profile was formed due to the surface tension.

### Experimental Setup

The TOF wrapped in an iron pipe was fixed on an adjustable five‐axis operating frame which can control the movement of the TOF in three dimensions with a resolution of 0.5 µm and the angular orientation of the TOF with a resolution of 0.5°. A rubber ring with a soap film was placed on a two‐dimensional moving stage with a resolution of 0.5 µm. The TOF operating frame was used to lower the tip of the TOF to a position about 20–30 µm away from the soap film surface, and a laser beam of 808‐nm wavelength from a continuous wave solid‐state laser (VLSS‐808‐B‐200, Shanghai Conet Laser Technology Co., LTD, China) was launched into the TOF to adjust and control the surface tension on the soap film. Real‐time images are recorded by a high‐speed CCD camera with computer interface.

### Transfer of Ordered Particle Structures onto Different Substrates

In the experiment, the assembled particle structures was placed in the center of the field of view under the microscope. Through the lifting platform, the PDMS substrate was slowly lifted at a speed of 0.5 mm s^−1^ until it approached the soap film. When PDMS touched the soap film, due to the viscosity of the soap film, the whole layer of soap film was adhered to the PDMS, and the formed particle structures on the soap film was also adhered to the substrate along with the soap film. Similarly, the transfer onto other substrates was realized.

## Conflict of Interest

The authors declare no conflict of interest.

## Author Contributions

H.X. and Z.H. conceived the idea, designed the experiments. Y.S. carried out the simulation. Z.H. and J.X. performed the experiments. H.X. and J.X. revised the manuscript. X.L., T.P., and G.Z. discussion the manuscript. H.X. and B.L. supervised the study.

## Supporting information

Supporting Information

Supplemental Video 1

Supplemental Video 2

Supplemental Video 3

Supplemental Video 4

## Data Availability

The data that support the findings of this study are available from the corresponding author upon reasonable request.
