# Supplementary material for: Opto‐Thermal‐Tension Mediated Precision Large‐Scale Particle Manipulation and Flexible Patterning
Source: Adv Sci (Weinh). 2024 Jul 25;11(36):2405211. doi: 10.1002/advs.202405211 (PMC11422809; doi:10.1002/advs.202405211)
Supplement: Supplementary file 1 — Supporting Information [file ADVS-11-2405211-s004.docx]

**Supporting Information**

**Opto-thermal-tension mediated precision large-scale particle manipulation and flexible patterning**

Ziyi He^1^, Jianyun Xiong^1^, Yang Shi^1^, Guoshuai Zhu^1^, Xing Li^1^, Ting Pan^1^, Baojun Li^1*^, and Hongbao Xin^1*^

^1^ Guangdong Provincial Key Laboratory of Nanophotonic Manipulation, Institute of Nanophotonics, Jinan University, Guangzhou 511443, China

**Email:** baojunli@jnu.edu.cn, [hongbaoxin@jnu.edu.cn](mailto:hongbaoxin@jnu.edu.cn)


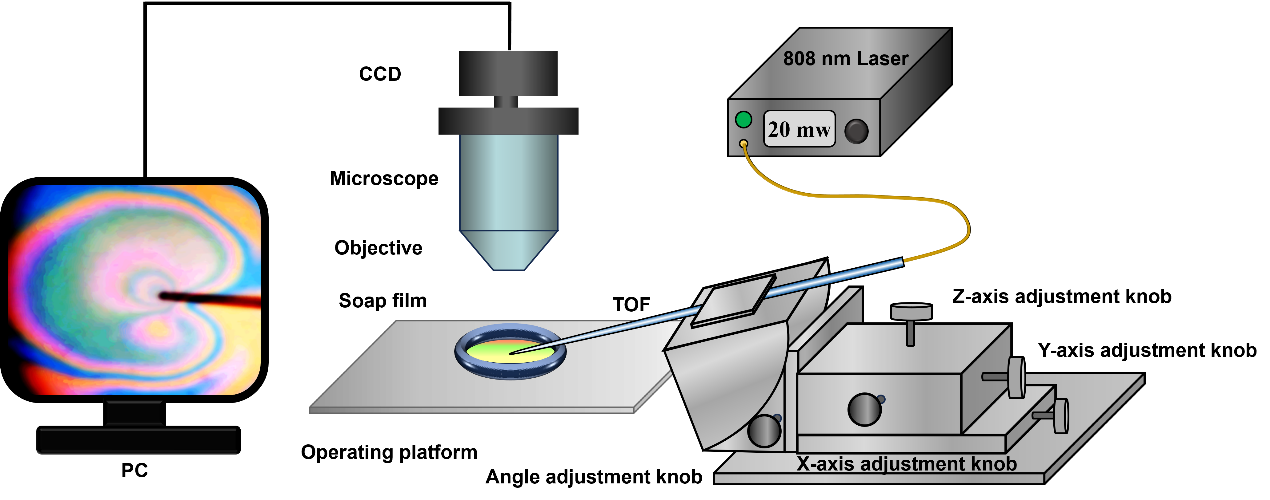


**Figure S1. Experimental setup.** A rubber ring with soap film was placed on a two-dimensional moving stage. A TOF was fixed on an adjustable five-axis operating frame and placed above the surface of a soap film with a distance of about 100 μm, and a laser beam of 808-nm wavelength from a continuous wave solid-state was launched into the TOF. Real-time images are recorded by a high-speed CCD camera with computer interface.


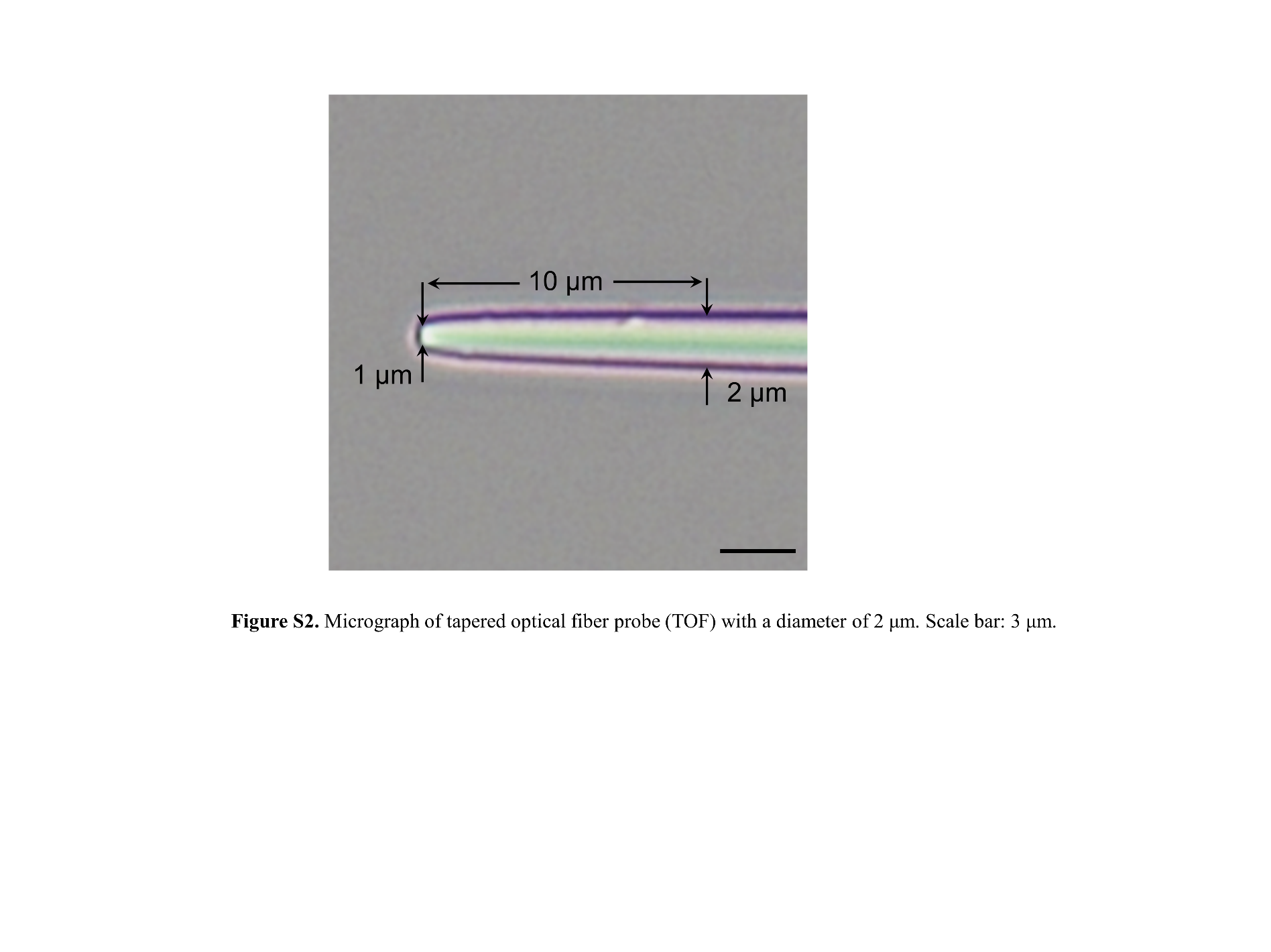


**Figure S2.** Microscopic image of tapered optical fiber probe (TOF) with a diameter of 2 μm. Scale bar: 3 μm.


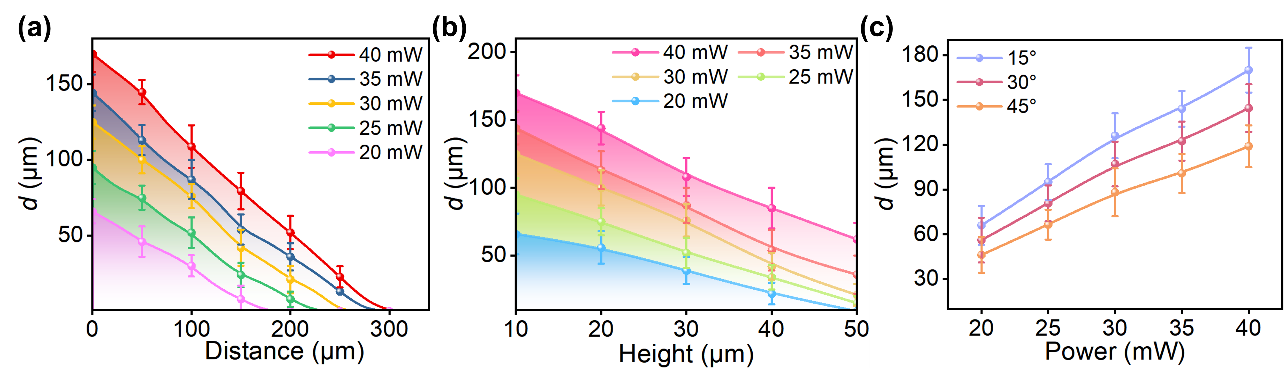


**Figure S3. Effective range for OTT regulation on soap film.** (a) The diameter of effect range of OTT as a function of distance between target and the tip of TOF. (b) The diameter of effect range of OTT as a function of height between soap film and the tip of TOF. (c) The diameter of effect range of OTT as a function of laser power and the angle of laser incident on soap film.


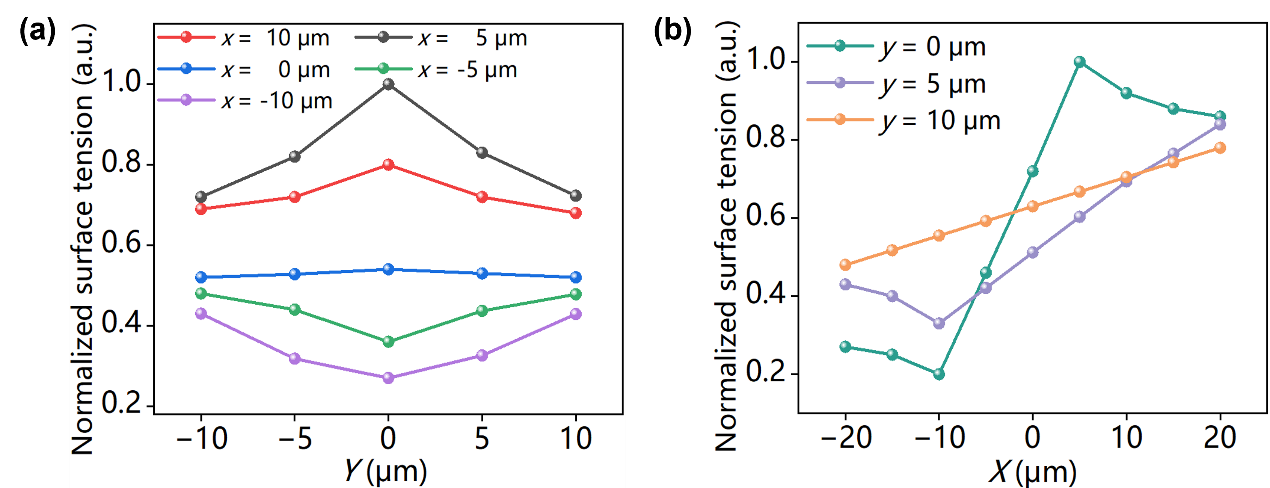


**Figure S4. Simulated normalized surface tension.** (a) Normalized surface tension as a function of the different position on the *x*-axis. The diagram showing the surface tension along the *y*-axis direction is symmetric about the *x*-axis. (b) Normalized surface tension as a function of the different position on the *y*-axis. The diagram showing the surface tension gradient along the *x*-axis.


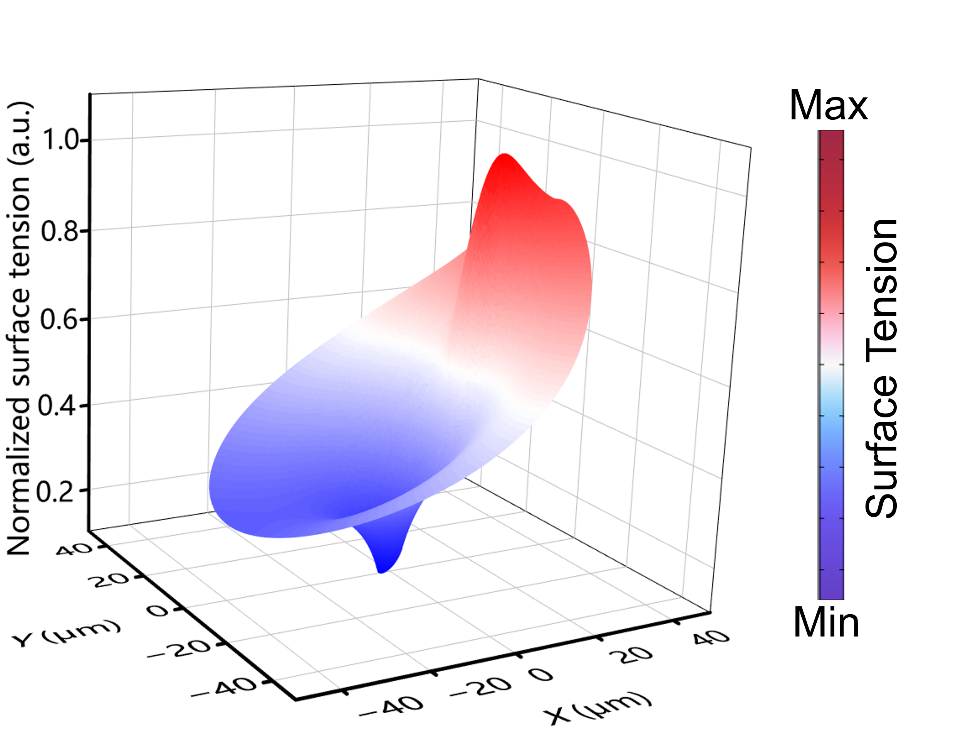


**Figure S5. Surface tension distribution of particle during movement.** The diagram showing the surface tension is symmetric about the *x*-axis.

As shown in Figure S6, there are six monodisperse particles in the field of view. A reference line was placed in the center of the field of view. Using our OTT method, the individual particles were controlled to move in *y*-axis direction and each particle's center was aligned accurately with the reference line. Eventually, the six initially randomly suspended particles were positioned along the reference line. The deviation between the center of each particle and the reference line was measured and analyzed separately. The measured data are -0.48, -0.22, -0.2, 0.4, 0.73, and 0.43 μm, respectively (Figure S6b). The calculated average absolute value of these six data points is ~0.45 μm, representing the displacement precision during the arrangement of the one-dimensional pattern.

**
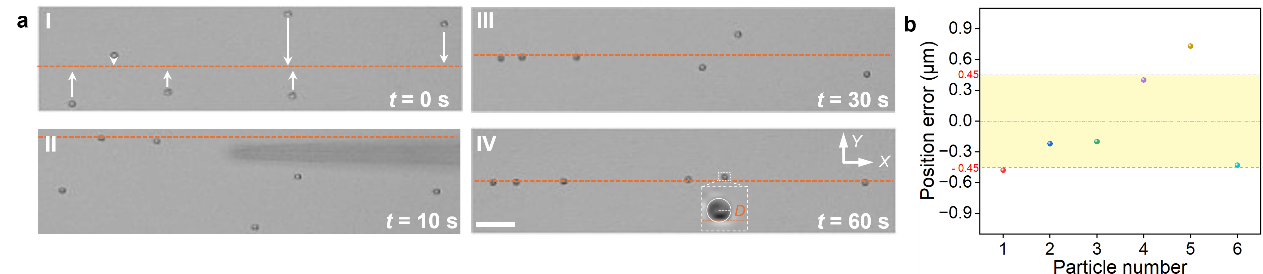
**

**Figure S6.** (a) Microscopic image showing the manipulation of six randomly suspended PS particles to a reference line (red line) along the *y* direction. White arrows indicate the direction of movement for each PS particle. The inset in panel IV defines the method used to determine the positional deviation D of individual PS particles. The orange line represents the reference line through the center of each PS particle. Scale bar: 10 μm. (b) Positional deviation of each PS particle in panel IV. The yellow shaded area represents the average deviation range between PS particles and the reference line, approximately 0.45 μm.

**
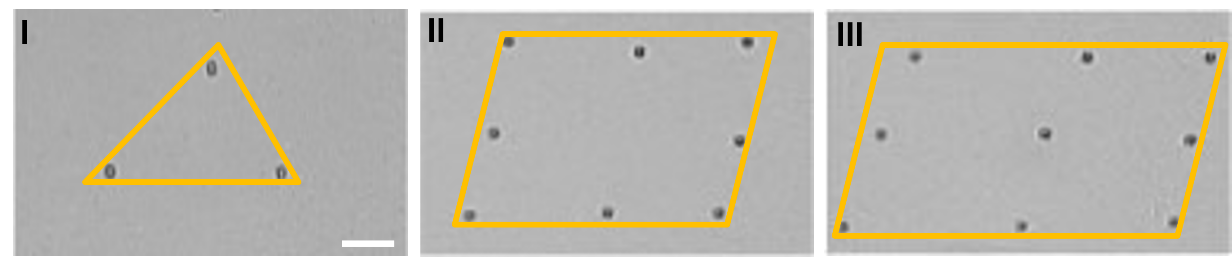
**

**Figure S7.** Manipulating individual particles to form discrete multi-particle structures of (I) 3-particle triangle, (II) 8-particle hollow parallelogram, and (III) 9-particle solid parallelogram. Scale bar: 10 μm.


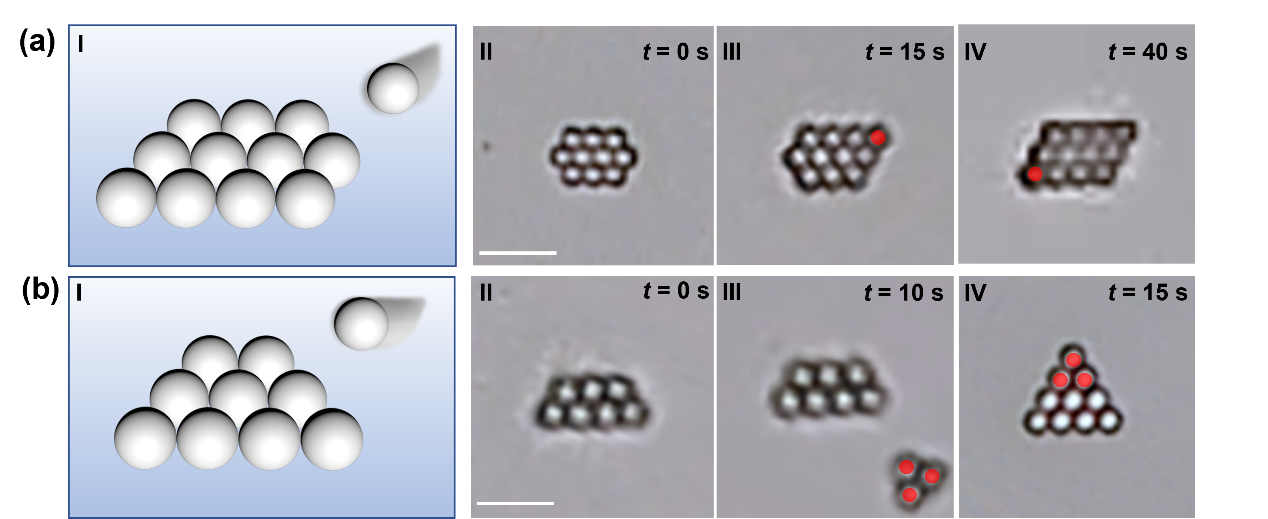


**Figure S8.** The OTT mediated assembly processes of (a) parallelograms and (b) triangles patterns. Scale bar: 10 μm.


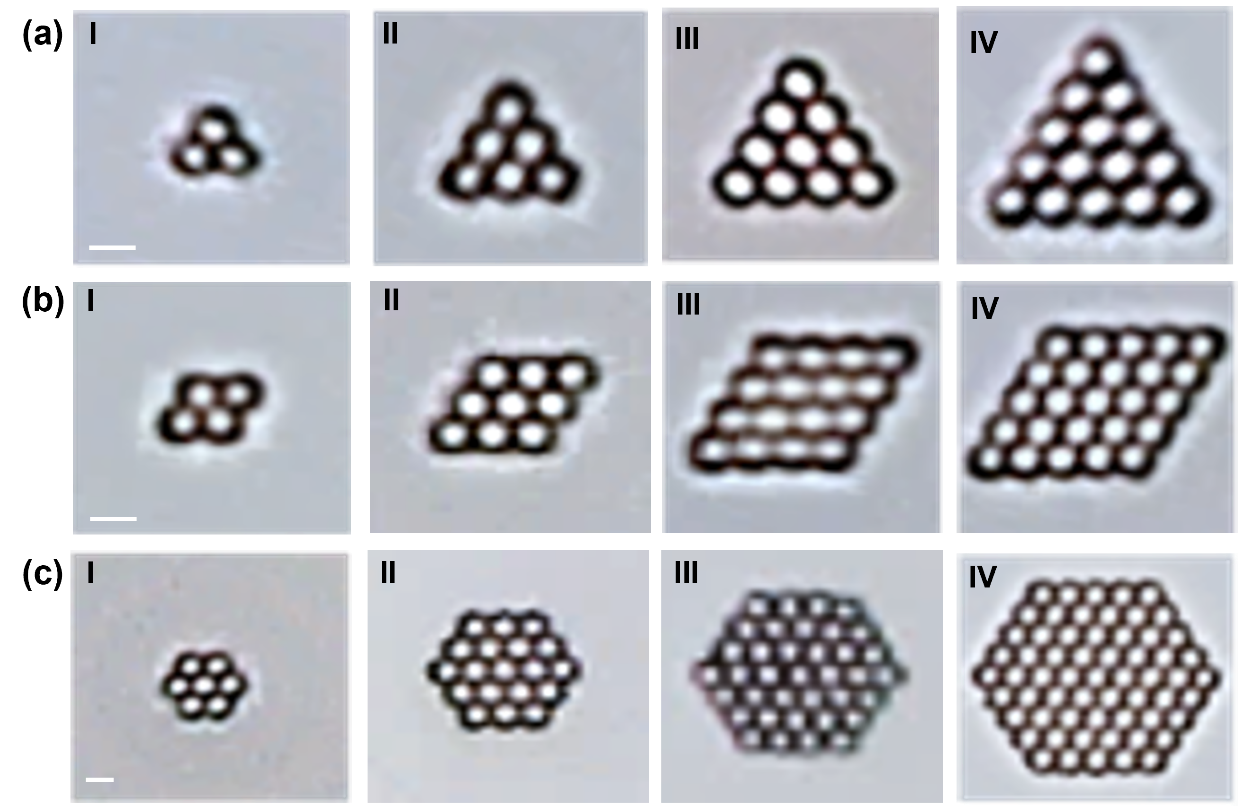


**Figure S9.** Assembly of regular patterns with increasing size, (a) regular triangle, (b) regular quadrilateral and (c) regular hexagon patterns. Scale bar: 5 μm.

**
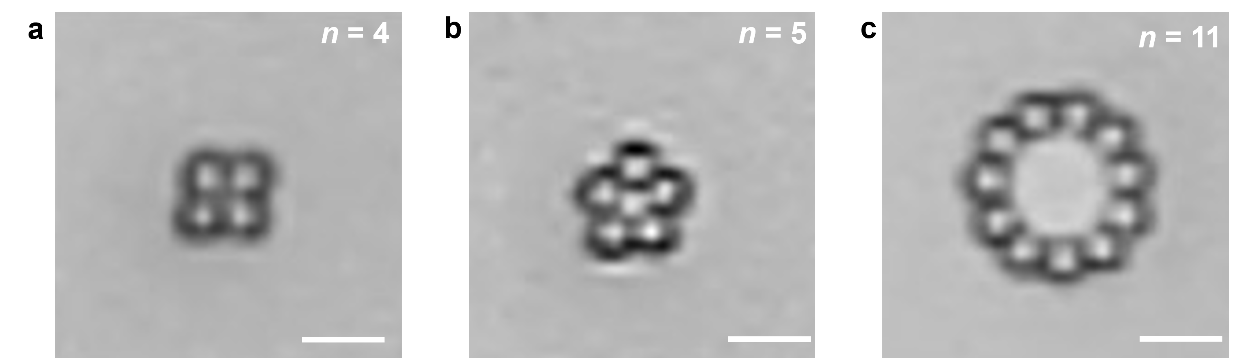
**

**Figure S10.** Patterning of non-close-packed structures on soap film. (a) Face-centered cubic structure assembled with 4 spherical 2-μm PS particles. (b) Pentagonal structure assembled with 5 spherical 2-μm PS particles. (c) Ring structure assembled with 11 spherical 2-μm PS particles. Scale bar: 5 μm.


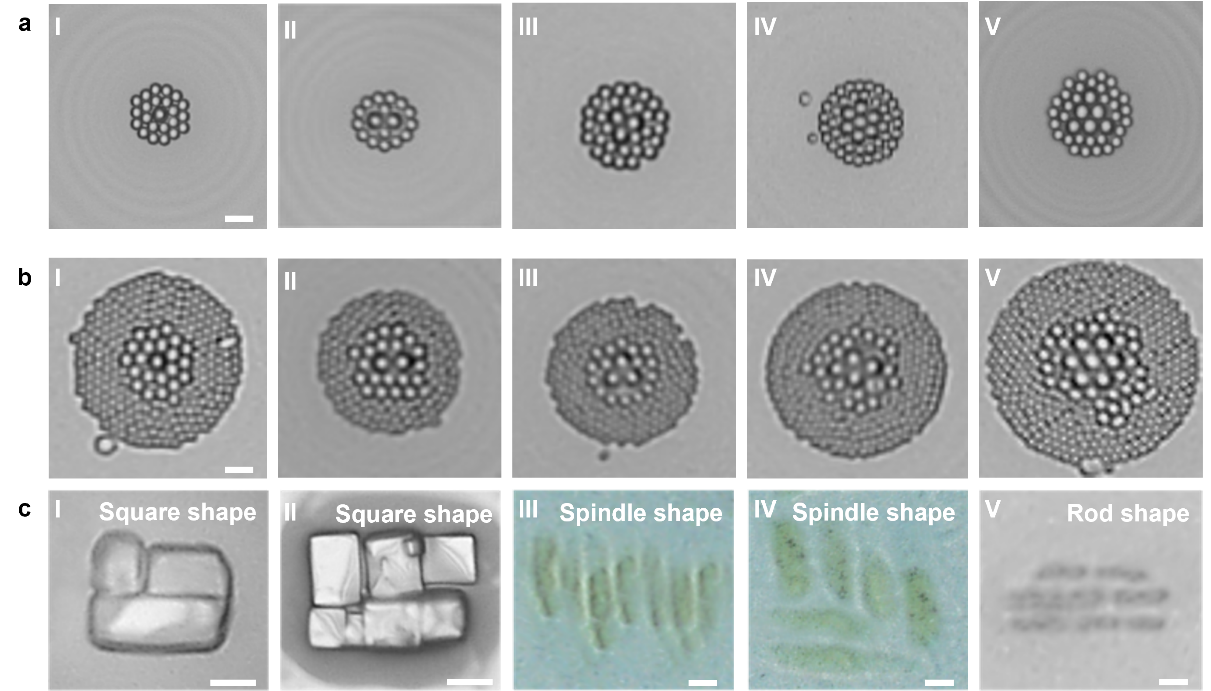


**Figure S11.** Hybrid patterning with different particles. (a) Hybrid patterning of different sized spherical microparticles with diameter of 3 and 2 μm. (b) Hybrid patterning of different sized spherical microparticles with diameter of 3, 2, and 1 μm. (c) Patterning different shaped particles of (I, II) square-shaped silica particles, (III, IV) spindle-shaped *Phaeodactylum tricornutum* Bohlin, and (V) rod-shaped *E. coli*. Scale bar: 5 μm.


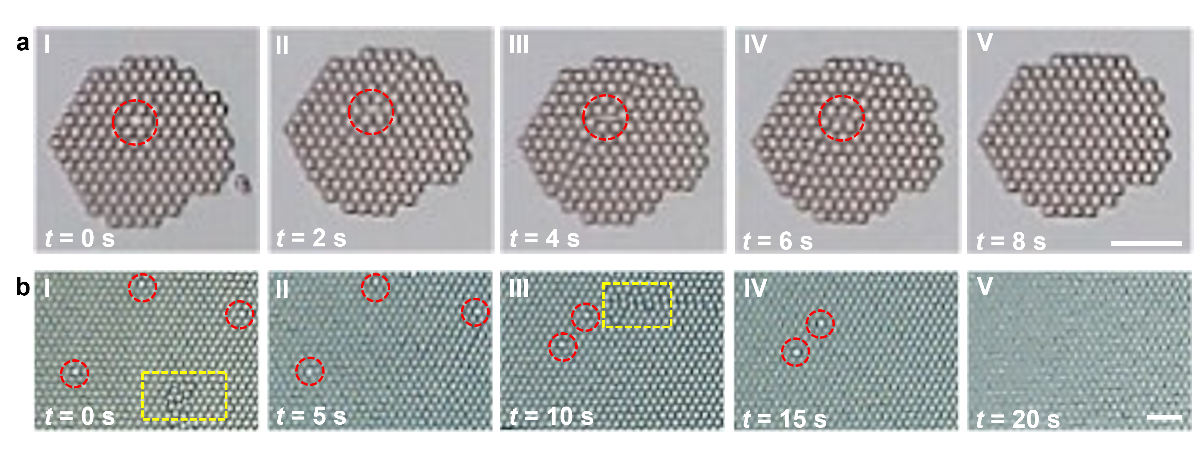


**Figure S12.** (a) Repairing of a single defect (vacancy) (dashed red circle indicated). (b) Repairing of multiple defects. Dashed red circles indicate the vacancy defects, while yellow dashed rectangular boxes indicate disordered particle defects. Scale bar: 10 μm.


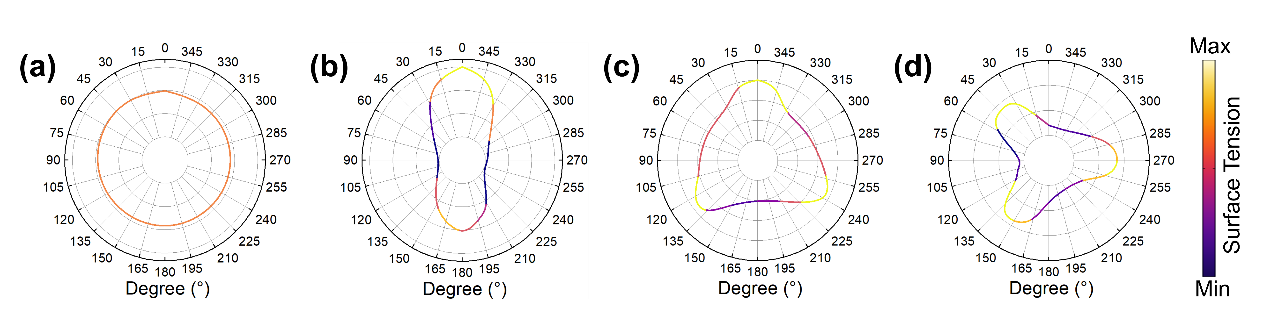


**Figure S13.** Surface tension field around different shapes of CCs. (a) Circle. (b) Ellipse. (c) Triangle. (d) Heart shape.


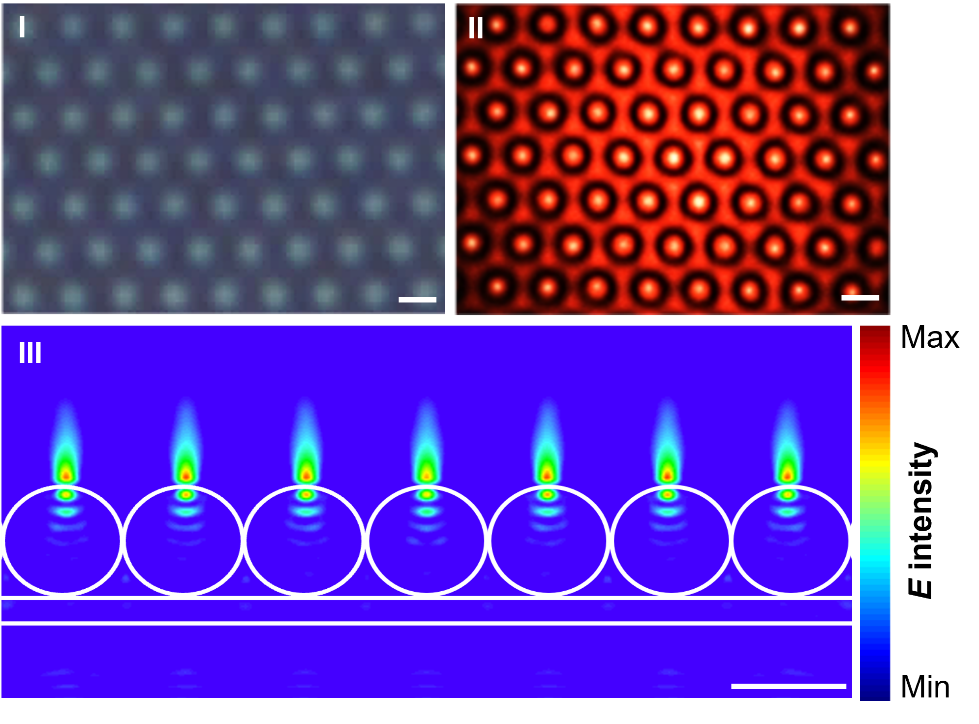


**Figure S14.** Large-scale photonic nanojet array generated by patterned particle structures. (I) Bright-field microscopic image of the patterned large-scale particle array. (II) Dark-field microscopic image showing light focusing effect by the large-scale microparticle-based microlens array. (III) Simulated focused light field due to the photonic nanojet effect by the microparticle-based microlens array. Scale bar: 3 μm.

**Supporting Video**

**Video S1:** Reconfigurable interference ring-Ellipse.

**Video S2:** Reconfigurable interference ring-Heart shape.

**Video S3:** Manipulation of single particle.

**Video S4:** Rotation of particle cluster.
